# Tolerant mechanism of model legume plant *Medicago truncatula* to drought, salt, and cold stresses

**DOI:** 10.3389/fpls.2022.847166

**Published:** 2022-09-07

**Authors:** Xiuxiu Zhang, Yu Sun, Xiao Qiu, Hai Lu, Inhwan Hwang, Tianzuo Wang

**Affiliations:** ^1^College of Biological Sciences and Biotechnology, Beijing Forestry University, Beijing, China; ^2^State Key Laboratory of Vegetation and Environmental Change, Institute of Botany, Chinese Academy of Sciencess, Beijing, China; ^3^Key Laboratory of Mollisols Agroecology, Northeast Institute of Geography and Agroecology, Chinese Academy of Sciencess, Changchun, China; ^4^Inner Mongolia Academy of Agricultural and Animal Husbandry Sciences, Hohhot, China; ^5^Department of Life Sciences, Pohang University of Science and Technology, Pohang, South Korea; ^6^College of Resources and Environment, University of Chinese Academy of Sciences, Beijing, China

**Keywords:** *Medicago truncatula*, abiotic stresses, morphological regulation, physiological regulation, functional genes, transcription factors

## Abstract

Legume plants produce one-third of the total yield of primary crops and are important food sources for both humans and animals worldwide. Frequent exposure to abiotic stresses, such as drought, salt, and cold, greatly limits the production of legume crops. Several morphological, physiological, and molecular studies have been conducted to characterize the response and adaptation mechanism to abiotic stresses. The tolerant mechanisms of the model legume plant *Medicago truncatula* to abiotic stresses have been extensively studied. Although many potential genes and integrated networks underlying the *M. truncatula* in responding to abiotic stresses have been identified and described, a comprehensive summary of the tolerant mechanism is lacking. In this review, we provide a comprehensive summary of the adaptive mechanism by which *M. truncatula* responds to drought, salt, and cold stress. We also discuss future research that need to be explored to improve the abiotic tolerance of legume plants.

## Introduction

The climate change products a series of environmental factors which show negative effects to plants (Farooq et al., 2022). Among the environmental constraints, drought, salt, and cold are the main abiotic stresses that influence plants’ physiological and biochemical processes, ultimately reducing crop production (Rhaman et al., 2021; Farooq et al., 2022). Up to 45% of the world’s farmland faces frequent water scarcity (Rhaman et al., 2021), and 20–50% of irrigated lands are affected by salinity (Munns and Tester, 2008). Approximately 57 and 26% of the world’s land and rural areas are affected by cold stress, respectively (Cramer et al., 2011). Much work has been devoted to explore the mechanism by which plants respond and adapt to abiotic stresses, and these findings have meaningful implications for improving crop production.

Legume plants are particularly important sources of food for both humans and animals. Abiotic stresses affect their growth and development. There is an eager need to clarify the mechanism by which legumes respond to abiotic stresses, and such research will aid the breeding of climate-resilient varieties. The legume model plant *M. truncatula* has small genome, short life cycle, self-pollination ability, and high genetic transformation efficiency (Tang et al., 2014). So, *M. truncatula* has been widely used in genomic, genetic, and physiological studies. Many studies focus on elucidating the mechanism by which *M. truncatula* responds and adapts to abiotic stresses. In this review, we summarize the general morphological, physiological, and molecular features by which *M. truncatula* responds and adapts to drought, salt, and cold stress. We also incorporate the crosstalk between different abiotic stresses, and discuss the implications for breeding stress-tolerant legume crops.

## Drought stress

**FIGURE 1:**
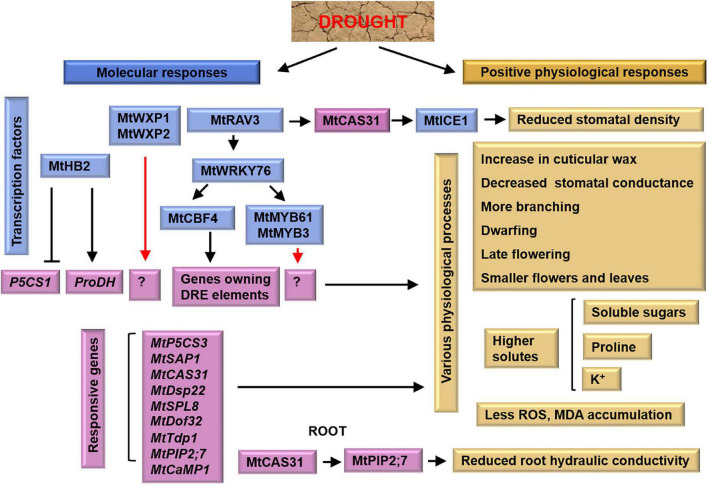
Tolerant mechanism of *M. truncatula* to drought stress. In shoots, MtRAV3 induces the expression of *MtCAS31*, whose encoded protein binds with MtICE1, resulting in reduced stomatal density. In roots, MtCAS31 facilitates the autophagic degradation of aquaporin MtPIP2; 7, and reduces root hydraulic conductivity. Blue boxes and purple boxes indicate transcription factors and responsive genes, respectively. Yellow boxes indicate physiological responses of *M. truncatula*. The black arrows represent the targets, the red arrows represent the unknown factors and the cross-lines mean suppression.

Drought stress significantly reduces leaf water potential and stomatal closure of *M. truncatula* plants, resulting in reduced photosynthesis, which in turn restricts plants’ growth (Nunes et al., 2008; Luo et al., 2016). Drought also causes photooxidative damage to thylakoid membranes and reduces chlorophyll content and photosystem II activity (Luo et al., 2016). To cope with drought stress, *M. truncatula* plants have evolved various responses such as alterations in tissue architectures and expression patterns of functional genes (Figure 1). Indeed, 5-week-old *M. truncatula* R108 plants are still recoverable after withholding irrigation for 12 days (Luo et al., 2016).

### Morphological and physiological regulation of drought tolerance

Plants’ shoot architecture is affected by drought stress and can be used as an indicator of drought adaptation (Farooq et al., 2009; Nguyen et al., 2013). Branching is a key determinant of shoot architecture. In *Arabidopsis*, AtSPL9 (squamosa promoter binding protein-like) controls the initiation of cauline leaf axillary meristems (Tian et al., 2014). In transgenic *M. truncatula* plants overexpressing *MtSPL8* inhibits branching by directly suppressing axillary bud formation (Gou et al., 2018). Down-regulation *MsSPL8* increases branch density and enhances drought and salt tolerance in transgenic alfalfa (Gou et al., 2018). Interestingly, *M. truncatula* plants with up-regulated *MtRAV3* (related to ABI3/VP1 transcription factor) have the similar tolerance to drought and salt stress, but exhibiting dwarfing, late flowering, and smaller leaves (Wang S. et al., 2021). Thus, MtRAV3 and MtSPL8 might play different roles in morphological development. However, Guo et al. (2021) find that overexpression of *MtDof32* (DNA-binding one zinc finger 32) in *Arabidopsis* results in reduced branches and enlarged leaves, but still have enhanced osmotic and salt tolerance. Although both *MtDof32* and *MtRAV3* enhance drought and salt tolerance in transgenic plants, they regulate different morphological development. Indeed, *MtDof32* enhances plants’ stress tolerance by regulating the rosette numbers. Thus, how to improve the shoot structure under drought condition might be a useful way to improve crops’ drought tolerance.

In addition, regulation of stomatal conductance (Nunes et al., 2008) and epidermal wax accumulation (Zhang et al., 2005) is an effective way for *M. truncatula* plants to cope with drought stress. Furthermore, osmotic and oxidative regulation are also essential in the response of *M. truncatula* to drought stress (Yousfi et al., 2010; Luo et al., 2016). For instance, *M. truncatula* populations adapt to drought tolerance by accumulating solutes such as proline, soluble sugars, and K^+^ (Yousfi et al., 2010). The *M. truncatula* lines overexpressing the oat arginine decarboxylase gene produce more soluble polyamines (PAs), resulting in greater drought tolerance compared to wild-type plants (Duque et al., 2016). The PAs including spermidine (Spd), spermine (Spm), and putrescine (Put) are involved in plant responses to abiotic stresses (Alcázar et al., 2010). Pagano et al. (2022) found that desiccation induces the expression of Spm synthase *MtSPMS* and Spd synthase *MtSPDS* in over-primed seeds. As for the oxidative reactions, *M. truncatula* plants that accumulate less peroxide and malondialdehyde (MDA) are more tolerant to drought stress (Luo et al., 2016; Wang S. et al., 2021).

### Molecular regulation of drought tolerance

#### Drought-related functional genes

Zhang et al. (2014) identified many drought-responsive genes in *M. truncatula*. The genes *MtP5CS* (encoding proline synthase) and *MtProDH* (encoding proline dehydrogenase) regulate proline accumulation coordinately in response to drought stress. Indeed, overexpression of *P5CS* in *M. truncatula* results in more proline accumulation and greater drought tolerance (Verdoy et al., 2006). Heterologous expression of calcium-binding protein gene *MtCaMP1* in *Arabidopsis* induces *P5CS1* and suppresses *ProDH*, making transgenic plants more tolerant to drought stress (Wang T. Z. et al., 2013). Whereas, plants with *Tnt1* transposon insertion of *MtP5CS3* accumulate less proline and are sensitivity to salt and drought stresses (Nguyen et al., 2013). In addition, cold-acclimation specific protein 31 (MtCAS31), a Y_2_K_4_-type dehydrin, interacts with AtICE1 (inducer of CBF expression 1) to regulate stomatal development. Overexpression of *MtCAS31* in *Arabidopsis* reduces stomatal density and significantly enhances drought tolerance in transgenic plants (Xie et al., 2012). Li et al. (2018) generate the *mtcas31* mutant by transcription activator-like effector nuclease (TALEN) technology, and identify that MtCAS31 interacts with leghemoglobin MtLb120-1 to regulate drought response. Moreover, in response to drought stress, MtCAS31 promotes the autophagic degradation of the aquaporin MtPIP2; 7, thereby reducing water loss and improving drought tolerance (Li et al., 2020). Recently, 39 autophagy−related (ATG) genes are identified in *M. truncatula*. Most of them are highly induced during seed development and drought stress, indicating that autophagy plays an important role in seed development and responses to drought stress in *M. truncatula* (Yang et al., 2021).

Plants overexpressing the stress-associated protein genes *MtSAP1* accumulate more nitric oxide (NO), which is beneficial to plant growth under osmotic and salt stress (Charrier et al., 2012, 2013). In turn, NO interacts with reactive oxygen species (ROS) to affect the *SAPs*’ expression (Delledonne et al., 2001; Wendehenne et al., 2004; Qiao and Fan, 2008). Of the 17 *MtSAPs*, the *MtSAP4*, *6*, *9*, *11*, *14*, and 15 are induced by drought stress (Zhou et al., 2018). In addition, Macovei et al. (2010) find that *MtTdp1*, a tyrosyl-DNA phosphodiesterase gene, is up-regulated by PEG treatment suggesting a relationship between drought response and DNA repair pathway. While, *MtTdp2*α positively regulates *M. truncatula* in salt response due to strong antioxidant effects of transgenic plants (Confalonieri et al., 2019). Recently, Pagano et al. (2022) found that desiccation treatment on over-primed seeds alters rRNA accumulation, promotes signal molecule 3′−phosphoadenosine 5′−phosphate (PAP) production, and up−regulates genes involved in ribogenesis. In addition, early light-inducible proteins (ELIPs) and ELIP-like proteins are pigment-binding components that protect against photooxidative damage (Araújo et al., 2013). Transgenic plants overexpressing the ELIP-like gene *CpDsp22* (desiccation stress protein 22 from *Craterostigma plantagineum*) recover faster from water deficit (Araújo et al., 2013). These results provide insights into NO and nucleic acid organization in response to oxidative stress caused by drought stress in *M. truncatula*.

#### Drought-related transcription factors

Transcription factors (TFs) regulate the transcription of downstream genes by binding to their *cis*-elements in promoters playing important roles in response to various stresses (Oztur et al., 2002; Porto et al., 2014). For instance, the C-repeat binding factor 4 (MtCBF4), belonging to the APETALA2/EREBP (AP2-EREBP) family, binds to the dehydration responsive (DRE) element of downstream genes to regulate drought response (Li et al., 2011; Table 1). Overexpression of TF *MtWRKY76* in *M. truncatula* promotes the expression of *MtCAS31*, *MtCBF4*, *MtMYB61*, and *MtMYB3*, and enhances drought tolerance in transgenic plants (Liu et al., 2016). Meanwhile, TF *MtRAV3* up-regulates the expression of *MtWRKY76*, *MtMYB61*, *MtCAS31*, *MtAOX1*, and *MtERF1* (Wang S. et al., 2021). In addition, the ethylene response factor (ERF) TFs MtWXP1 and MtWXP2 mediate cuticular wax production. Overexpression of *MtWXP1* and *MtWXP2* enhances transgenic plants’ drought tolerance (Zhang et al., 2005, 2007). These two wax genes are expected to have great potential for crop improvement through genetic modification. While, TF MtHB2 is a homeodomain leucine zipper (HD-Zip) protein that negatively regulates drought stress by affecting osmotic and oxidative responses (Song et al., 2012). Li et al. (2022) identifies 15 *HD-ZIP ?* genes in *M. truncatula*. In particular, *MtHB7* and *MtHB12* are positively associated with salt, osmotic stress, and abscisic acid (ABA), while *MtHB13* and *MtHB23* are negatively associated with these stresses. This genome-wide analysis of the HD-ZIP I TFs in *M. truncatula* provides valuable references for further research.

**TABLE 1:** TFs of *M. truncatula* involved in drought, salt, and cold stress.

Family	Transcription factors	Downstream genes	Stress	References
AP2/EREBP	MtCBF1	Unknown	Cold	Pennycooke et al., 2008; Zhang et al., 2011; Sun et al., 2021
	MtCBF2	Unknown	Cold	
	MtCBF3	Unknown	Cold	
	MtCBF4	*MtCAS15, MtCOR15A, MtCOR15B, MtKIN1, MtRD17, MtRD29A, MtRD29B*	Drought, salt, cold	Li et al., 2011; Zhang et al., 2016; Sun et al., 2021
	MtDREB1C	Unknown	Cold	Chen et al., 2010
	MtWXP1	Unknown	Drought, cold	Zhang et al., 2007
	MtWXP2	Unknown	Drought, cold	
MYB	MtMYB3	*MtCBF4*	Cold, drought	Zhang et al., 2016
	MtMYB61	*MtMYB3*	Cold, drought	
	MtMYBS1	*AtP5CS*	Salt	Dong et al., 2017
	MtMYB634	Unknown	Salt	Gruber et al., 2009
	MtMYB636	Unknown	Salt	
	MtMYB119	Unknown	Salt	
	MtMYB1070	Unknown	Salt	
bHLH	MtbHLH-658	Unknown	Salt	Zahaf et al., 2012
WRKY	MtWRKY76	Unknown	Drought, salt	Liu et al., 2015
TFIIIA-like	MtZpt2-1	*MtCorA1, MtFpf1, MtPrp2*	Salt	Merchan et al., 2007
	MtZpt2-2	Unknown	Salt	
HD-ZIP	MtHB1	*MtLBD1*	Salt	Ariel et al., 2010
	MtHB2	*AtP5CS1, AtProDH*	Drought, salt	Song et al., 2012
	MtHB7, MtHB12, MtHB13, MtHB23	Unknown	Drought, salt	Li et al., 2022
NAC	MtNAC969	Unknown	Salt	de Zélicourt et al., 2012
RAV	MtRAV3	*MtWRKY76, MtMYB61, MtCAS31, MtAOX1, MtERF1*	Drought, salt	Wang S. et al., 2021

#### Drought-related plant growth regulators

Plant growth regulators (PGRs) such as auxin, ABA, and ethylene regulate plants in response to abiotic stresses (Rhaman et al., 2021). Both PEG and ABA treatment induces the expression of 9-*cis*-epoxycarotenoid dioxygenase gene *NCED5* leads to increased endogenous ABA content in *M. truncatula* (Planchet et al., 2011; Luo et al., 2016). Meanwhile, water deficit induces endogenous NO accumulation through an ABA-dependent pathway (Planchet et al., 2014). While, exogenous ABA addition induces asparagine and proline production contributing to osmotic adjustment under water deficit (Planchet et al., 2011). However, the modulation of proline metabolism is independent of NO production under water deficit (Planchet et al., 2014). So, exploring the central role of ABA in water-deficit tolerance could lead us to obtain more information on osmotic adjustment and nitrogen metabolism under adverse conditions.

## Salt stress

**FIGURE 2:**
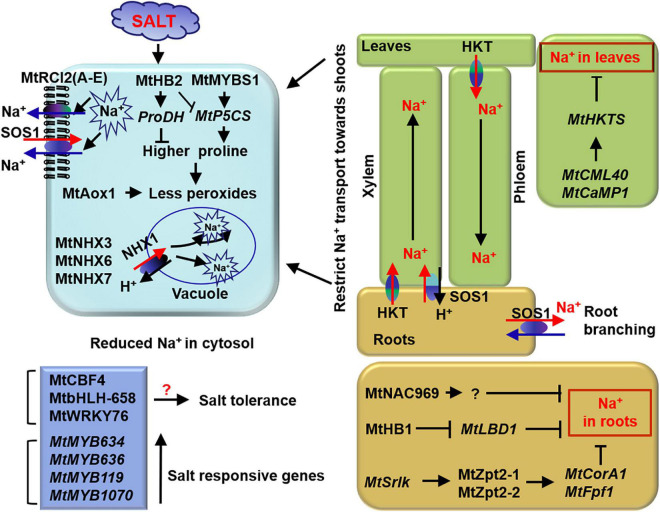
Tolerant mechanism of *M. truncatula* to salt stress. MtHB2 and MtMYBS1 and oxidase MtAox1 are involved in osmotic regulation. Membrane proteins MtRCI2 (A–E) and Na^+^/H^+^ exchangers MtNHX3, MtNHX6, and MtNHX7 play roles in reducing Na^+^ accumulation in cells. MtNAC969, MtHB1, MtZpt2-1, and MtZpt2-1 play roles in reducing Na^+^ accumulation in roots. While, Ca^2+^ sensor MtCML40 and MtCaMP1 participate in Na^+^ long-distance transportation regulation between roots and shoots. Light blue box represents cells. Blue boxes represent transcription factors related to salt stress. Green and yellow boxes mean shoots and roots, respectively. The black arrows represent the targets or directions, the red arrows represent transport of Na^+^ and the cross-lines mean suppression.

Salt stress causes osmotic stress, ion toxicity, and oxidative damage to *M. truncatula* plants, resulting in reduced photosynthesis and biomass (Yousfi et al., 2010; Arraouadi et al., 2012; Luo et al., 2016; Gou et al., 2018; Zhang X. X. et al., 2019; Wang S. et al., 2021). *M. truncatula* minimizes these damages by regulating the production of osmolytes and antioxidants in cells, the extrusion of Na^+^ out of cells, and the reduction of Na^+^ in leaves (Figure 2). In fact, hydroponic *M. truncatula* R108 can tolerate 100 mM NaCl for nearly 1 week (Merchan et al., 2003; Zhang X. X. et al., 2019;). *M. truncatula* genotype TN1.11 is the most tolerant to salt stress among R108, Jemalong A17, TN6.18, and TN1.11.

### Morphological and physiological regulation of salt tolerance

The root architecture of plants is affected by salt stress. de Zélicourt et al. (2012) find that shorter and less branched roots are beneficial for preventing Na^+^ uptake in *M. truncatula* (Ariel et al., 2010).

Multiple studies show that *M. truncatula* plants with high drought tolerance also display strong salt tolerance, suggesting some overlapping mechanism between them (Yousfi et al., 2010; Luo et al., 2016; Gou et al., 2018; Wang S. et al., 2021). Both salt and drought responses involve osmotic and oxidative regulation. López et al. (2008) find that accumulation of amino acids and sugars in shoot cells alleviates the adverse effects of Na^+^ in *M. truncatula*. Salt-tolerant *M. truncatula* genotypes accumulate more antioxidants and have strong peroxide scavenging ability (Mhadhbi et al., 2011, 2013; Amouri et al., 2018). In rice seedlings, PAs regulate cell membrane stability as ROS scavengers and antioxidants under salt stress (Ghosh et al., 2012). The sensitive *M. truncatula* cultivar TN6.18 has a lower (Spd + Spm)/Put ratio, indicating that this ratio may be related to oxidative status (Antoniou et al., 2021). Nevertheless, the PA levels are easily affected by plants’ condition and environment. So, the application of (Spd + Spm)/Put ratio and PA levels to asses salt tolerance in plants needs to be determined.

Salt stress causes ion toxicity in plant cells. Greater Na^+^ accumulation in *M. truncatula* leads to smaller root and shoot structures (Arraouadi et al., 2012; Zhang X. X. et al., 2019). When *M. falcata* and *M. truncatula* are subjected to salt shock, *M. falcata* shows stronger tolerance for its effective extrusion of Na^+^ out of cells (Liu et al., 2015). This result is in agreement with that *M. truncatula* lines with the highest salt stress tolerance have the lowest Na^+^ content in leaves (Aydi et al., 2008). In addition, legumes tend to restrict Na^+^ transport toward the shoots to keep a relatively low Na^+^ content in their photosynthetic organs (Winter and Läuchli, 1982). Transgenic *M. truncatula* lines overexpressing the calmodulin-like gene *MtCML40* are more sensitive to salt stress because of the greater Na^+^ accumulation in their shoots (Zhang X. X. et al., 2019).

### Molecular regulation of salt tolerance

#### Salt-related functional genes

Several salt-responsive genes throughout leaf senescence are identified in *M. truncatula* (Dong et al., 2021). These genes are mainly related to protein and amino acid metabolism, photosynthesis, chlorophyll metabolism, and hormone signaling. Long et al. (2016, 2018) characterize the proteome-level changes associated with the salt stress response, which are consistent with previous studies (Kang et al., 2010; Dong et al., 2021). Hence, *M. truncatula* responds to salt stress by altering gene expression, biosynthesis of proteins and metabolites, and modifications in hormonal signaling, etc.

Several studies show that proline is related to the regulation of salt stress. *Arabidopsis* plants expressing *MtHB2* are susceptible to salt stress due to lower proline and soluble sugar content in the cells. This is because MtHB2 may bind to the promoters of *P5CS1* and *P5CS2* to inhibit their expression (Song et al., 2012). Besides, De Lorenzo et al. (2009) identify a salt-induced gene *Srlk* in *M. truncatula* and RNA interference (RNAi) created *Srlk* mutants accumulate less Na^+^ in plants than in wild-type plants. Liu et al. (2015) find that the SOS (salt overly sensitive) system mediates cytosolic Na^+^ out of cells. The protein MtCaMP1 up-regulates the vacuolar Na^+^/H^+^ antiporter *AtNHX1* and reduces Na^+^ content in transgenic *Arabidopsis* plants (Wang T. Z. et al., 2013). The NHX transporters sequester Na^+^ into vacuoles and decrease the Na^+^ damage to the organelles in the cytoplasm. Four distinct NHX isoforms (AtNHX1–AtNHX4) are confirmed in Arabidopsis, and their roles in vacuolar ion and pH homeostasis have been determined (Bassil et al., 2019). In *M. truncatula*, six *MtNHX*s are identified, and *MtNHX3*, *MtNHX6*, and *MtNHX7* in roots are induced by salt stress (Sandhu et al., 2018). In addition, Du et al. (2021) identify several salt stress responsive *CBL-CIPK* genes in *M. truncatula* and *M. sativa.* Collectively, *SOS* pathway, *CBL-CIPK* family genes, and *NHX* genes play crucial roles in response to salt stress.

#### Salt-related transcription factors

Gruber et al. (2009) identify many salt-responsive TFs in *M. truncatula* roots belonging to AP2/EREBP, HD-ZIP, and MYB families (Table 1). TF MtMYBS1 promotes the expression of *P5CS* and mitigates the restriction of root growth under salt stress (Dong et al., 2017). TF MtHB1 suppresses the expression of *MtLBD1* (lateral organ boundaries gene), reducing lateral roots formation and Na^+^ uptake (Ariel et al., 2010). Furthermore, overexpression of *MtNAC969* induces the formation of shorter and less branched roots, whereas RNAi-mediated *MtNAC969* inactivation promotes lateral root formation. Interestingly, both root systems improved plant growth under salt stress (de Zélicourt et al., 2012). This discrepancy might because that MtNAC969 might participate in multiple pathways controlling root system adaptation to salt stress. In addition, overexpression of *MtbHLH-658*, *MtRAV3*, and *MtWRKY76* improves root growth under salt stress in transgenic plants (Zahaf et al., 2012; Liu et al., 2016; Wang S. et al., 2021). Merchan et al. (2007) identify two salt responsive IIIA-like TFs MtZpt2-1 and MtZpt2-2. Overexpression each of them significantly improves root growth under salt stress (De Lorenzo et al., 2007). There exists many stress-related *cis*-elements in *MtZpt2-1*, allowing it to respond and adapt to abiotic stresses (Wang T. Z. et al., 2014). However, the target genes for most of these TFs have not been identified.

#### Salt-related epigenetic regulation

Epigenetic modifications play important “switch” roles in regulating gene expression, thereby affecting plant responses to abiotic stresses (Dong et al., 2018). The epigenetics refers to alterations in gene expressions caused by DNA methylation and histone modification (Saeed et al., 2022). Yaish et al. (2018) analyze the *M. truncatula* genome-wide DNA methylation in response to salt stress and find that the whole DNA methylation level is increased, and the 5-methylcytosine nucleotide (5-mC) landscape is remodeled under salt stress. More precisely, the DNA methylation and histone modification of *MtMYBS1* are analyzed under salt stress. Indeed, the expression of *MtMYBS1* is negatively correlated with its DNA methylation modification, and positively correlated with histone H3K9ac modification under salt stress (Dong et al., 2018). These studies provide critical theoretical guidance for further understanding of epigenetic regulation in response to salt stress in *M. truncatula*.

#### Salt-related plant growth regulators

Bianco and Defez (2009) compared *Mt-RD64* plants noduled by *Sinorhizobium meliloti* RD64, which have higher indole-3-acetic acid (IAA) content in nodules and roots, with the control plants. The results show that *Mt-RD64* plants accumulate higher endogenous osmolyte in shoots and are more tolerant to salt stress (Bianco and Defez, 2009). Thus, exogenous IAA might be able to stimulate osmolyte production and positively affect plant development and differentiation under salt stress.

## Cold stress

**FIGURE 3:**
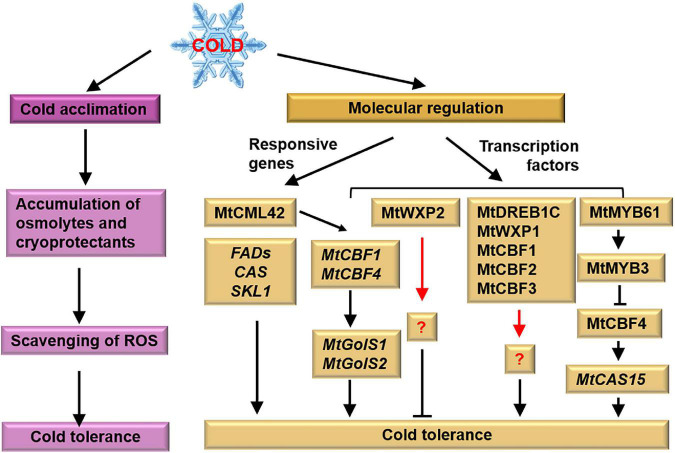
Tolerant mechanism of *M. truncatula* to cold stress. *M. truncatula* enhances its cold tolerance after cold acclimation. Meanwhile, cold responsive genes and transcription factors associated with cold tolerance have been identified. Pink and yellow boxes represent physiological and molecular responses, respectively. The black arrows represent the targets, the red arrows represent the unknown factors and the cross-lines mean suppression.

Cold stress includes chilling stress and freezing stress. When the temperature is low but above 0°C (i.e., chilling), membrane fluidity decreases. When it is below 0°C and is defined as freezing, ice formation might occur within tissues, resulting in membrane damage (Raza et al., 2021). The freezing tolerance of many plants is increased after exposure to low, non-freezing temperatures, which is referred to as cold acclimation (Xin and Browse, 2000; Figure 3). Cold acclimated *M. truncatula* A17 seedlings exposed to −10°C are still survivable (Zhang et al., 2011). In molecular terms, the cold responsive genes and CBF-dependent signaling pathways play roles to enhance the cold tolerance of *M. truncatula* (Figure 3).

### Morphological and physiological regulation of cold tolerance

Cold-acclimated *M. truncatula* exhibits shorter stems, fewer leaves, smaller tissues, and higher root/shoot ratio compared to plants under normal condition (Thapa et al., 2008). While, the relationship between these phenotypes and cold tolerance remains vague. Pennycooke et al. (2008) find that cold acclimation does not significantly improve the survival rate of *M. truncatula* under freezing stress. However, Thapa et al. (2008) and Zhang et al. (2011) demonstrate that cold acclimation is able to improve the freezing tolerance of *M. truncatula*. The different cold acclimation regimes, 4°C in Zhang et al. (2011) and 2°C in Pennycooke et al. (2008), and different cultivars and ages of seedlings may explain this discrepancy. Thapa et al. (2008) propose that 3.5°C day/–1°C night for 1 week is the best regime for cold acclimation in *M. truncatula*. Cold acclimation induces the accumulation of sucrose and proline in *M. falcata* and *M. truncatula* (Zhang et al., 2011). Although *MtP5CS3* is induced at 4°C, it is unlikely associated with cold tolerance (Nguyen et al., 2013). These data indicate that the cold tolerance might be independent with the proline concentration but positively correlated with the soluble sugar concentration.

### Molecular regulation of cold tolerance

#### Cold-related functional genes

Zhang et al. (2018) figure out 20 *MtFAD* genes (fatty acid desaturase genes) involved in chilling response. The *FADs* are involved in the desaturation of fatty acids affecting the function of the membrane system (Wallis and Browse, 2002). Another important agent of cold-related genes is the cold-responsive (*COR*) genes. Mohapatra et al. (1989) isolate three *COR*s specifically expressed during cold acclimation in *Medicago* referred to as *CAS* (cold acclimation specific). The *CAS18* gene of *M. falcata* is positively correlated with freezing tolerance and its expression is much higher in cold-acclimated plants than in non-acclimated plants (Wolfraim et al., 1993). Pennycooke et al. (2008) find that the *M. truncatula* genome contains a single *CAS31* gene, whereas the *M. falcata* genome contains multiple *MfCAS30* and *MfCAS31* genes. So, *M. falcata* is more tolerant to cold stress than *M. truncatula*. Zhang et al. (2011) clarify that *MfCAS17* and *MfCAS18* contribute to the stronger cold acclimation effects on *M. alfalfa* than on *M. truncatula*. Zhao et al. (2014) find that cold acclimation—induced the transcription of *MtCAS15* is suppressed in the ethylene-insensitive mutant *skl*, indicating that *MtSKL1* is required for cold acclimation.

#### Cold-related transcription factors

Recently, the expression profiles of *DREBs* in *M. truncatula* and *M. sativa* are identified in the cold-stress response (Shu et al., 2016; Sheng et al., 2022). A cluster of *DREB* subfamily members on *M. truncatula* chromosome 6 is induced by both cold and freezing stress (Shu et al., 2016), and 33 *MsDREBs* are significantly upregulated by cold treatment (Sheng et al., 2022). The genome-wide identification of *DREBs* in *Medicago* species provides promising molecular targets for the improvement of cold tolerance in crops. Overexpression of *MtDREB1C/MtCBF3* inhibits shoot growth and enhances the freezing tolerance of *M. truncatula* (Chen et al., 2010). Transgenic *M. truncatula* plants overexpressing *MfERF1* show enhanced tolerance to both freezing and chilling stress through promoting PA turnover, antioxidant protection, and proline accumulation (Zhuo et al., 2018). Overexpression of *WXP1* in *M. truncatula* enhances the plants’ freezing tolerance without altering growth and development. However, plants overexpressing *WXP2* are more sensitive to freezing (Zhang et al., 2007). These results indicate that *WXP1* is a useful candidate gene for improving plant freezing tolerance by genetic conduction.

The TFs MtCBF1, MtCBF2, and MtCBF3 have been shown to participate in cold acclimation in *M. truncatula* (Pennycooke et al., 2008; Zhang et al., 2011). TF MtCBF4 not only positively regulates cold acclimation and freezing tolerance but also enhances drought and salt tolerance (Li et al., 2011; Zhang et al., 2016). Although the differential response of MtCBFs to cold stress is unknown, the major components involved in CBF-dependent signaling pathways are illustrated under cold stress. TF MtMYB3 binds to the *cis*-elements of *MtCBF4* promoter and represses its expression. TF MtCBF4 directly activates the transcription of *MtCAS15*. TF MtMYB61 relieve the inhibitory effect of MtMYB3 on *MtCBF4* (Zhang et al., 2016). Besides, Qu et al. (2016) indicates that the MfNAC3 plays roles in response to cold stress by regulating the expression of *MtCBF4*. Recently, Sun et al. (2021) identifies that MtCML42 positively regulates the expression of *MtCBF1* and *MtCBF4*, thereby upregulating the expression of the *COR* genes, *MtGolS1* and *MtGolS2*, and leads to raffinose accumulation and improved cold tolerance.

#### Cold-related epigenetic regulation

Demethylases containing Jumonji (JMJ) C domain are involved in removal of methyl groups at lysine or arginine residues (Lu et al., 2008). In *M. truncatula*, *MtJMJC5* undergoes cold-specifically induced alternative splicing, which is reversible depending on temperature (Shen et al., 2016). Previous studies show that AtJMJ30/JMJD5 is a component of the plant circadian clock (Lu et al., 2011). So, there may exist a *MtJMJC5*-dependent link between the circadian clock and ambient temperature fluctuation in *M. truncatula*.

#### Cold-related plant growth regulators

Zhao et al. (2009) proposes that nitrate reductase (NR)-dependent NO production plays an important role in the cold acclimation-induced increase in freezing tolerance by modulating proline accumulation in *Arabidopsis*. In addition, the role of NO in cold acclimation through the regulation of glutathione (GSH) synthesis has been studied in *M. falcata* and *M. truncatula* (Zhang P. P. et al., 2019). Exogenous application of ethylene reduces cold acclimation-induced freezing tolerance (Zhao et al., 2014). These results indicate that there may have some relationships between NO and ethylene molecules and osmotic regulation in response to cold tolerance.

## Conclusion and future perspectives

**FIGURE 4:**
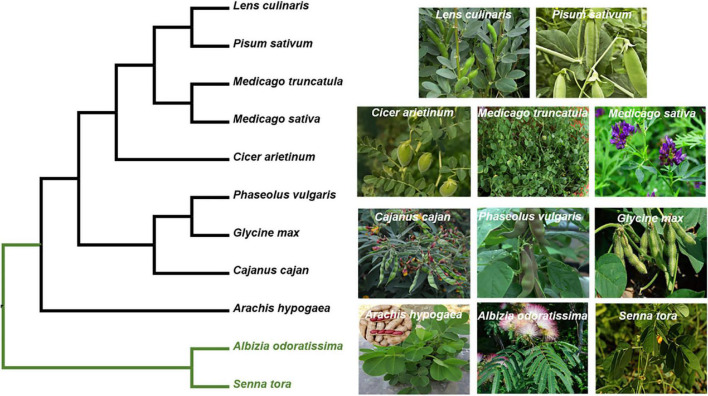
Phylogeny of *M. truncatula* with the other legumes. The phylogeny of the targeted species was reconstructed based on their plastomes. The data matrix of Azani et al. (2017) was used as a basic DNA matrix, from which we extract the sequences of the targeted species and outgroups (*Albizia odoratissima* and *Senna tora*). Then, these sequences were aligned with the complete plastomes of *M. truncatula*. The maximum likelihood (ML) phylogeny was reconstructed using RAxML version 8.2.12. Their accession number obtained from GenBank. *Albizia odoratissima*: NC_034987.1; *Arachis hypogaea*: NC_026676.1; *Cajanus cajan*: NC_031429.1; *Cicer arietinum*: NC_011163.1; *Glycine max*: NC_007942.1; *Lens culinaris*: NC_027152.1; *Medicago sativa*: KU321683.1; *Medicago truncatula*: JX512024.1; *Phaseolus vulgaris*: NC_009259.1; *Pisum sativum*: NC_014057.1; *Senna tora*: NC_030193.1.

**FIGURE 5:**
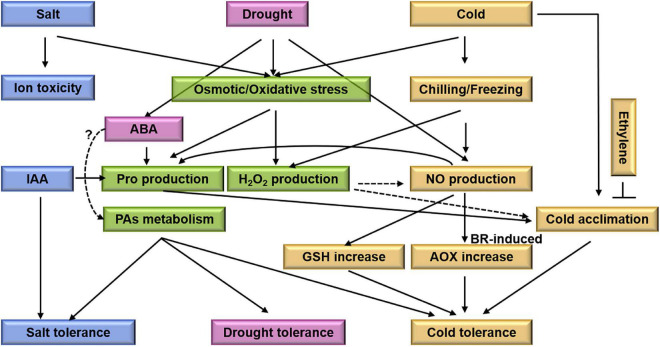
A schematic representation of cross-talks between drought, salt, and cold stress in *M. truncatula*. Drought, salt, and cold stress cause secondary stress including osmotic stress and oxidative stress. And then induce proline and H_2_O_2_ production. In *M. truncatula*, ABA could induce proline accumulation, contributing to osmotic adjustment under drought condition. And NO in cold acclimation through regulation on GSH synthesis and redox is associated with the differential cold tolerance. Meanwhile, ethylene reduces cold acclimation-induced freezing tolerance. IAA may have positive effects on their development and differentiation under salt stress. PAs as compound positively regulate *M. truncatula* in response to drought, salt, and cold stress. Ion toxicity and cold acclimation are specific process for salt stress and cold stress, respectively. Blue boxes indicate salt related elements. Pink and yellow boxes indicate drought and cold related elements, respectively. Green boxes indicate the central elements between drought, salt, and cold stress. The black arrows represent the targets, the dotted lines represent the possible regulatory targets and the cross-lines mean suppression.

Legumes are a particularly important source of food for livestock worldwide (Wang T. Z. et al., 2021). The conventional breeding of crops is time-consuming, labor-intensive, and cost-inefficient (Mishra et al., 2021). An efficient solution is to generate stress-tolerant varieties with the help of information obtained in the lab. Thus, understanding the physiological and molecular processes of legumes in response to abiotic stresses is really important. *M. truncatula* is closely related to many legumes and forages (Figure 4). In this review, we summarize the mechanism by which *M. truncatula* responds and adapts to drought (Figure 1), salt (Figure 2), and cold stress (Figure 3) as well as crosstalk between them (Figure 5). These studies provide genetic resources and molecular markers that could be used in future studies.

All drought, salt and cold stress can induce osmotic and oxidative stress. Proline is involved in abiotic tolerance as a compatible osmolyte, molecular chaperone, and ROS scavenger (Szabados and Savouré, 2010). There are three *MtP5CS*s in *M. truncatula*. The *MtP5CS1* is constitutively expressed and the *MtP5CS3* participates in osmotic regulation (Armengaud et al., 2004; Kim and Nam, 2013). Both IAA and ABA induce proline accumulation but enhance the salt and drought tolerance, respectively, indicating that proline play different roles in salt and drought stress. Meanwhile, both drought and cold stress induce NO production, however, drought or cold induced NO production *via* different pathways (Planchet et al., 2014; Arfan et al., 2019). Complex crosstalk suggests that plants integrate hormones and signaling pathways to get better adaptation to abiotic stresses. With the help of modern molecular technologies, such as transgenic and CRISPR/Cas9 approaches, clarifying the functions of different factors is promising. Recently, Wang et al. (2022) using the CRISPR/Cas9 toolkit generates single and double knockout mutants in *MtDMP8* or *MtDMP9* and assesses their roles in haploid induction in *M. truncatula*. However, there is no report about tolerant mechanism to abiotic stresses using CRISPR/Cas9 technology.

Currently, many studies on *M. truncatula* are carried out in the culture room. So, more field experiments should be performed in future research. In field condition, plants often face several distinct environmental stresses simultaneously. For example, plants in arid regions often suffer from drought and heat stress (Iyer et al., 2013). In *M. truncatula*, interactive effects of ozone and drought have been well studied (Iyer et al., 2013). However, how combined occurrence of other kinds of abiotic stresses impact growth and development of *M. truncatula* is still not known yet and will be an important research topic in the future. In addition, *M. truncatula* is a cultivated species, some stress tolerance genes might have been lost during the domestication process (Wang T. Z. et al., 2021). *M. ruthenica*, a wild *Medicago* forage, retains these genes. Therefore, *M. ruthenica* provides a valuable model plant for studying the molecular mechanism of abiotic stresses tolerance in legumes.

## Author contributions

XZ and TW conceived the concept of the work and wrote the manuscript. YS, XQ, HL, and IH revised the manuscript. All authors approved the final manuscript.
